# Interactions between Medical Plant-Derived Bioactive Compounds: Focus on Antimicrobial Combination Effects

**DOI:** 10.3390/antibiotics11081014

**Published:** 2022-07-28

**Authors:** Natalia Vaou, Elisavet Stavropoulou, Chrysoula (Chrysa) Voidarou, Zacharias Tsakris, Georgios Rozos, Christina Tsigalou, Eugenia Bezirtzoglou

**Affiliations:** 1 Laboratory of Hygiene and Environmental Protection, Department of Medicine, Democritus University of Thrace, Dragana, 68100 Alexandroupolis, Greece; empezirt@yahoo.gr; 2 Centre Hospitalier Universitaire Vaudois (CHUV), 1101 Lausanne, Switzerland; 3 Department of Agriculture, School of Agriculture, University of Ioannina, 47100 Arta, Greece; xvoidarou@uoi.gr (C.V.); clevervet@hotmail.com (G.R.); 4 Laboratory of Microbiology, Department of Medicine, National and Kapodistrian University of Athens, 11527 Athens, Greece; ztsakris@gmail.com; 5 Laboratory of Microbiology, Department of Medicine, Democritus University of Thrace, Dragana, 68100 Alexandroupolis, Greece; xtsigalou@yahoo.gr

**Keywords:** medicinal plants, bioactive compounds, antimicrobial combination effects, synergy, antagonism, metabolomics, challenges, future perspectives

## Abstract

It is accepted that the medicinal use of complex mixtures of plant-derived bioactive compounds is more effective than purified bioactive compounds due to beneficial combination interactions. However, synergy and antagonism are very difficult to study in a meticulous fashion since most established methods were designed to reduce the complexity of mixtures and identify single bioactive compounds. This study represents a critical review of the current scientific literature on the combined effects of plant-derived extracts/bioactive compounds. A particular emphasis is provided on the identification of antimicrobial synergistic or antagonistic combinations using recent metabolomics methods and elucidation of approaches identifying potential mechanisms that underlie their interactions. Proven examples of synergistic/antagonistic antimicrobial activity of bioactive compounds are also discussed. The focus is also put on the current challenges, difficulties, and problems that need to be overcome and future perspectives surrounding combination effects. The utilization of bioactive compounds from medicinal plant extracts as appropriate antimicrobials is important and needs to be facilitated by means of new metabolomics technologies to discover the most effective combinations among them. Understanding the nature of the interactions between medicinal plant-derived bioactive compounds will result in the development of new combination antimicrobial therapies.

## 1. Introduction

Research in medicinal plant-derived mixtures tends either to focus on one or two bioactive compounds (secondary metabolites) or to ignore the chemical composition altogether, studying the biological effects of complex mixtures for which bioactive compounds are unknown. Moreover, research design may be complicated by the chemical complexity and variability of the medicinal plant extracts/bioactive compounds [1,2,3,4]. Recent studies to elucidate the mechanisms of action of traditionally used medicinal plant extracts have found a complex mixture of bioactive compounds that act at multiple targets [5,6,7,8].

Most of the studies presenting antimicrobial properties of medicinal plant extracts and bioactive compounds make use of unfractionated extracts that usually show weak in vitro antimicrobial activity. These studies were infrequently confirmed by means of in vivo assays. Thus, the precise mechanisms of action of the vast majority of such bioactive compounds are unknown [9].

The scientific search for medicinal plant extracts is challenging because of their huge complexity and variability. Since complex plant extracts and no single bioactive molecules are often used for medicinal purposes, understanding the interactions between the active compounds could be of great importance [10,11,12]. It was found that disease resistance is less likely to occur against a combination of bioactive compounds than against single active molecules [11,12,13]. Medicinal plants are targeting microbes through the combined action of structurally and functionally diverse active compounds. Combined effects can vary based on the target microbial species [10,14].

Medicinal plant extracts may contain hundreds or even thousands of individual bioactive compounds in varying abundances and identifying the bioactive compounds responsible for a given biological activity is a significant challenge [15]. In fact, the overall activity of medicinal plant extracts is a result of the combined action of multiple compounds with synergistic, additive, or antagonistic activity [12,16,17,18,19]. It is not acceptable to use bioactive compounds as medicines without understanding intra-extract interactions on a wider scale. In several instances, medicinal plant extract activity shows a better effect than an equivalent dose of an isolated compound [20] and cannot be predicted on what is known about individual compounds. Whether it is synergy, enhanced bioavailability, cumulative effects, or simply the additive properties requires further research.

Synergistic interactions between the compounds of individuals or mixtures or medicinal plant extracts are a vital part of their therapeutic efficacy. Therefore, medicinal plant extract synergy needs to be evaluated by rigorous analysis methods and validated in clinical trials. Moreover, the specific bioactive compounds responsible for those effects and the basic mechanisms by which they interact are still incompletely understood [21].

The main objective of this critical review is to present an update of the published data that currently exist to understand antimicrobial combination effects, including both synergy and antagonism, within complex mixtures of plant-derived bioactive compounds. In particular, existing approaches that have developed significantly in the last years are highlighted for studying synergistic or antagonistic antimicrobial combinations of bioactive compounds and elucidating mechanisms and underlying their interactions. It is questionable whether the practice of isolating, purifying, and concentrating compounds is really the best way to study and apply medicinal plants or if this will be proved to be worthless or harmful. Challenges of combination effects, study limitations, and perspectives are also analyzed. Finally, this review aims to provide practical information to researchers who are evaluating the bioactive compounds and mechanisms responsible for the antimicrobial combination effects of complex mixtures.

## 2. Terminology of Combination Effects

Several articles were published to provide valuable commentary on the definition of combination effects in complex mixtures [10,11,12]. Although the evaluation of interactions between multiple bioactive compounds is popular [20,22,23], it remains difficult to give an accurate definition for the term synergy [7,22,23,24,25] since there are present compounds about which we know very little, either chemically, pharmacologically or even quantitatively [12,26,27]. However, it is generally agreed that interactions between multiple bioactive compounds can be classified as synergistic, additive, or antagonistic. 

**Figure 1:**
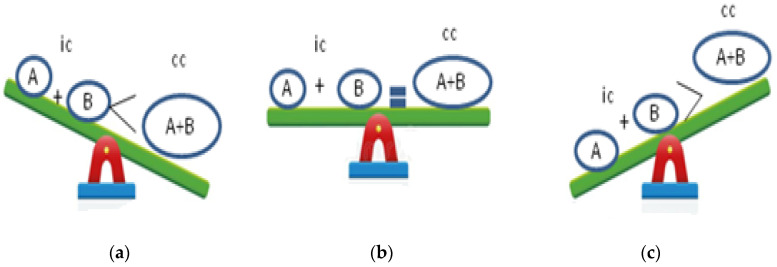
Combination effects of bioactive compounds: (**a**) synergistic effect; (**b**) additive effect; (**c**) antagonistic effect; ic: individual compounds; cc: combined compound.

Synergy occurs when the combined effect of compounds is greater than the sum of their individual effects. Synergy may also arise when one compound enhances the therapeutic effect of another compound by regulating its absorption, distribution, metabolism, and excretion or when all compounds involved are inactive on their own but become active when combined. Additive and non-interactive combinations indicate that the combined effect of the two compounds is a pure summation effect, while an antagonistic interaction results in less than the sum of the effects of the individual compounds. However, the additive effect is not simply the sum of the effects of compound A plus B, but it is computed from the individual effects based on a complex mathematical algorithm equation. Antagonism is much easier to define, being a reduced effect from that which is expected [28,29,30,31] (Figure 1).

## 3. Medicinal Plants as Complex Systems

Complex systems science is an interdisciplinary approach to science offering an understanding of collective behaviors of whole systems representing complex integrations of interconnected subsystems or parts linked to their environments [32]. Complex systems have multiple structures and functions existing in relationship with information networks and information exchange. The integration in complex systems creates a system that is greater than any isolated part [33].

Using complex systems science, plants as living organisms may be considered dynamic, self-organizing, and environmentally adaptive. Therefore, medicinal plants are complex adaptive systems. Complex adaptive properties emerge from the chemical compounds of each medicinal plant. It is the chemical matrix that provides a profile of plant complexity [34]. Chemical analysis is required not only to establish a correlation between complex mixtures and molecular interaction properties, but also to understand the putative synergy, complex cellular processes, and biochemical pathways through the metabolite-to-gene network [35]. Moreover, plant complexity exists to the greatest degree within the intact state of the plant and this complexity is reduced the further away from the natural state that the plant moves.

### 3.1. Medicinal Plants and Synergy

Potentiating plant synergy occurs because the interactive effects of the plant chemical matrix, characterized by plurality and diversity of chemical compounds, are greater than the additive effects of individual compounds. This synergy modulates the biochemical pathways and changes the membrane potentials, receptor selectivity, and protein shifts [36]. 

Attenuating synergy arises when compounds in one plant bind to compounds in another to offer protection from toxicity. Physiological synergy occurs when plant compounds act together to enhance and facilitate absorption, bioavailability, and metabolism and in turn, decrease potential adverse effects [37].

### 3.2. Medicinal Plants and Nonlinear Therapeutic Causality

Therapy with medicinal plants is a nonlinear, indirect process that emerges from engagement with complex networks within a system. The nonlinear therapeutic causality is demonstrated when local and small perturbations from medicinal plants result in widespread non-specific effects, which may be unexpected, disproportionate, and non-sequential in relation to the cause [38]. Medicinal plant compounds act through interactive mechanisms of adaptation to stress at different levels and the human organism mediates patterns of response as a result of network interactions. This may also explain how medicinal plants act to perturb therapeutic patterns on self-organization [39].

## 4. Antimicrobial Activity of Medicinal Plant-Derived Compounds

Plants are complex, adaptive, and synergistic systems [40]. The low frequency of infectious diseases found in wild plants, in contrast to crop plants, is due in part to the synergistic effects of multiple antimicrobial compounds [41]. Plants are also known to have co-evolved with microorganisms and thus established effective chemical responses [42]. Plants and bacteria share a “genetic instability” and thus they may respond to environmental stressors by rearranging their genotype [43]. 

Chemical complexity and the multi-targeted nature of medicinal plants are considered therapeutic strengths but make the identification of compounds a difficult target [44]. Moreover, medicinal plant extracts of combinations of compounds show a better antimicrobial effect than isolated compounds. A combination of non-specific mechanisms of action might create a more effective antimicrobial than an antibiotic. Additionally, studies have shown that plant-derived antimicrobials often do not induce resistance. It remains to be understood if those antimicrobials will be subject to the same antimicrobial resistance as the existing antibiotics [45,46,47].

## 5. Antimicrobial Combination Effects of Medicinal Plant-Derived Mixture Compounds

The uniqueness of medicinal plants is due to their use in combinations and to the interactions between bioactive compounds. Synergy, a key factor in medicinal plant medicine, is an effect seen by a combination of compounds being greater than would have been expected by adding together their separate contributions [48]. Proving and defining synergy is difficult since complexity in methodology in identifying interaction effects exists. To do this, the testing of individual compounds and comparing the activity with an equivalent dose in the mixture is necessary. Although some studies confirm this methodology, the term “polyvalent” is used to denote an improved and cooperative effect without qualifying it [49].

It is generally agreed that the use of a combination of multiple antimicrobial agents can result in different combined effects depending on the composition and concentration of the compounds. Specifically, synergy is obtained when two antimicrobial compounds are combined and produce antibacterial activity greater than the sum of the antibacterial activity of the individual compound. An additive effect is produced by combining antimicrobials producing an antimicrobial activity that is equal to the sum of the individual compound. An antagonist effect results when the antimicrobial activity of two compounds in combination is less than the sum of the effects of the individual compounds [50,51].

Testing antimicrobial combinations for their interactive effects helps to attain the following purposes: (1) synergistic combinations could be discovered and enhance antimicrobial effects of the individual compounds; (2) it would exclude antagonistic interactions which can be harmful; and (3) it would minimize the toxicity and adverse effects of the compounds since they could be used in lower doses in the combination than when used individually [52].

## 6. Reported Examples of Synergistic/Antagonistic Antimicrobial Combination Effects

Whole plant preparations are more effective than isolated compounds due to the interactions between compounds within them [53]. Considerable evidence exists that combination effects within medicinal plant extracts can alter the antimicrobial activity of a mixture. Nevertheless, the vast majority of complex medicinal plant-derived compounds still await chemical research considering that the combined effect of medicinal plant extracts may be complicated [54]. Here, we provide some examples in which synergistic and antagonistic antimicrobial activity with medicinal plant extracts were documented.

Recent studies on antimicrobial complex mixtures of bioactive compounds of essential oils (EOs) have mainly reported synergistic or antagonistic effects rather than additive effects. In addition, several studies showed that EOs have an antimicrobial activity stronger than their main compounds individually tested [55]. For example, the combination of eugenol with linalool or menthol exhibited the strongest synergy, suggesting that the combination of monoterpenoid phenol with monoterpenoid alcohol is effective [56]. Moreover, the antibacterial synergy between eugenol and three other bioactive compounds (cinnamaldehyde, carvacrol and thymol against *Escherichia coli*) was reported [57]. A synergistic effect was also found between carvacrol and thymol against *Penicillium* spp., *Aspergillus flavus,* and *Fusarium* species with fractional inhibitory concentration (FIC) index ≤ 0.05. Thyme and oregano EO combined showed synergistic effects against different fungal species with FIC index < 0.5, except for *Aspergillus niger*, which exhibited an additive effect with an FIC index of 0.75 ± 0.16. However, a synergistic effect was exhibited by combined peppermint and tea tree EO against *Aspergillus niger* [58,59,60].

Propolis’s antimicrobial activity has been widely studied and its antibacterial and antifungal activity was demonstrated [61]. It is known that propolis’s antibacterial activity results from flavonoids, phenols, aromatic acids, and sesquiterpenes [62]. The three main flavonoids found in propolis, namely pinocembrin, chrysin, and galangin, were shown to possess combination effects. In a study, the interactive efficacy of these three compounds against nine micro-organisms was investigated (Table 1). From the 27 combinations searched, six combinations (22%) were synergistic and nine combinations (33%) were additive. The Gram-positive and Gram-negative microbes had four additive interactions each, but the fungal pathogens showed only one additive interaction. 

*Listeria monocytogenes* was the microorganism most sensitive to the compound combinations. The remaining 45% of the combinations were non-interactive, but no antagonism was found. The interaction between galangin and chrysin exhibited synergy regardless of the ratio at which they were mixed [63]. Evaluating the activity of polyphenols mixture against *Staphylococcus aureus***,** it was found that the concentrations of the compounds in the mixture were lower than the minimum inhibitory concentration (MIC) of the most active compound used separately. This result supports the hypothesis that propolis’s antimicrobial activity is attributed to the synergistic action of several compounds rather than the presence of one compound of high antimicrobial activity [64].

*Hydrastis canadensis* L. (Ranunculaceae) contains several flavonoids, alkaloids, and other compounds with diverse structures. It was found that the flavonoids from *Hydrastis canadensis,* 6-desmethyl-sideroxylin, 8-desmethyl-syderoxylin and sideroxylin enhance through synergy the antibacterial activity of the alkaloid berberine against *Staphylococcus aureus* by acting as bacterial efflux pump inhibitors (EPIs). However, several alkaloids contained in *Hydrastis canadensis* do not act as EPIs or exhibit biologically relevant antibacterial activity [65,66,67].

Artemisinins, produced by *Artemisia annua* L. (Asteraceae), are accepted as safe and potent antimalarial agents [68]. Both in vitro and in vivo studies showed that various combinations including artemisin and its derivatives could be utilized as antimalarial therapies. Two compounds with antiplasmodial activity, artemisitene and 9-epi-artemisinin, were found to antagonize the efficacy of artemisinin against both chloroquine-sensitive and chloroquine-resistant strains. However, some compounds identified within the extract did not exhibit the same combination effect at all concentrations evaluated. For example, 3-caffeoylquinic acid showed an additive effect in combination with artemisinin at a ratio of 1:3 (artemisinin: 3-caffeoylquinic acid) when evaluated against the chloroquine-sensitive strain, but at higher ratios (1:10–100), synergistic interactions were identified. Moreover, the flavone casticin exhibited antagonistic activity at a 1:3 ratio but was reported to enhance the in vitro activity of artemisinin by 3–5-fold in other studies using higher combination ratios (1:10–1.000) [69,70,71].

## 7. Synergistic Interactions between Compounds and Antibiotics

Combining antibiotics with activity-enhancing plant-derived compounds is a significant strategy in the rapidly developing antimicrobial therapy options. Certain medicinal plants can inactivate antibiotic resistance mechanisms. This ability is due to synergy between medicinal plant compounds and antibiotics whose activity would be low when compounds are absent. In addition, compounds show relevant activity only when they are utilized together with an antibiotic. However, identifying which compound in an extract is responsible for the synergistic interaction is difficult. Additionally, some compounds show synergistic activity by other mechanisms, in addition to their own antimicrobial activity due to “polyvalent” effects [48,49].

### 7.1. Essential Oils

EOs and their bioactive compounds comprise part of the group of secondary metabolites that can interact with antibiotics. A combination of five EOs with seven antibiotics was studied; the combined effect of peppermint, cinnamon bark, and lavender EO with piperacillin and meropenem showed significant synergy against different *Escherichia coli* strains [72]. 

The combination of *Origanum compactum,* *Chrysanthemum coronarium*, *Melissa officinalis*, *Thymus willdenowii,* Boiss, and *Origanum majorana*, EOs with gentamycin, tobramycin, imipenem, and ticarcillin against ten Gram-positive and Gram-negative bacterial strains showed synergy in some cases, but also an antagonistic effect against different bacterial strains was found [73]. In a recent study, EOs prepared from *Laurus nobilis* L. and *Prunus armeniaca* L. species were tested for potential synergistic antibacterial and antifungal effects with three antibiotics, namely fluconazole, ciprofloxacin, and vancomycin. The EO from *Laurus nobilis* had the highest antimicrobial activity, with MICs ranging from 1.39 to 22.2 mg/mL for bacteria and between 2.77 and 5.55 mg/mL for yeasts. Of the 32 interactions evaluated, 23 (71.87%) exhibited total synergy, and nine (28.12%) a partial synergy. The main EOs from *Laurus nobilis* (eucalyptol, a-terpinyl acetate, and methyl eugenol) showed the highest synergistic effect with all the antibiotics tested with FIC index values in the range of 0.266 to 0.75 for bacteria, and between 0.258 and 0.266 for yeasts [74].

In another study, the investigation of combinations in *Eucalyptus camaldulensis* EOs with three conventional antibiotics (gentamycin, ciprofloxacin, and polymyxin B) exhibits synergy even in some re-sensitized multi-drug-resistant *Acinetobacter baumannii* strains. The detected MICs for the *Eucalyptus camaldulensis* EOs were in the range from 0.0005 to 0.002 mg/mL. When two *Eucalyptus camaldulensis* Eos were combined with ciprofloxacin, synergy was identified against two out of three tested multi-drug-resistant *Acinetobacter baumannii* strains with an FIC index value <0.5 [75]. Bioactive compounds of Eos, namely thymol and carvacrol, exhibited synergy with penicillin against *Escherichia coli* and *Salmonella typhimurium*. In addition, carvacrol was found to exhibit synergy in combination with both ampicillin and nitrofurantoin against *Klebsiella oxytoca***,** with FIC index values of 0.375 and 0.15, respectively, while thymol was non-active. Carvacrol showed the highest MIC values of 2.5 mg/mL against *Klebsiella oxytoca* [76]. It was found that eugenol exhibited synergy with ampicillin against *Streptococcus cricetid* and *Streptococcus gordonii* and with gentamycin against *Streptococcus sanguinis* and *Porfyromonas gingivalis.* The MIC for eugenol was found to be between 0.1 and 0.8 mg/mL in combination with eugenol and ampicillin the MIC was reduced >4–8-fold in all tested bacteria producing synergy as defined by the FIC index ≤0.375–0.5 [77]. The antibacterial and streptomycin-modifying activity of the *Thymus glabrescens* EO was also studied. The main compounds of this EO were geraniol, geranyl acetate, and thymol. The MIC for *Thymus glabrescens* EO was identified to be between 2.508 and 5.0168 mg/mL. All the combinations studied between compounds and streptomycin showed mainly antagonistic interactions. Combinations between geraniol and thymol produced a dominant additive effect (FIC index 0.76 to 1.09) [78]. 

Phenols, alcohols, and oxygenated monoterpenes identified in some EOs are effective in biofilm destruction [79]. Rosato et al. [80] reported the synergistic effect of *Cinnammonum zeylanicum, Mentha piperita*, *Origanum vulgare*, and *Thymus vulgaris* EOs with gentamycin (FIC index 0.08 to 0.16), oxacillin (FIC index 0.08 to 0.23) and norfloxacin (FIC index 0.08 to 0.23) on bacterial biofilm growth of four different strains of Gram-positive bacteria. The interaction of EOs with norfloxacin was the most effective on biofilm growth in all the tested combinations. 

### 7.2. Propolis

Propolis is a complex biological mixture. Row propolis usually contains 50–70% plant resins, 30% waxes, 10% EOs and aromatic oils, 5% pollen, and 5% other organic bioactive compounds. Bioactive compounds include flavonoids (flavanols, flavones, and flavanones), aromatic acids, terpenes, esters, aldehydes, coumarin, sterols, and fatty acids [81].

The synergy between propolis and other antibiotics can potentially prevent resistance, increase antibacterial efficacy and provide wider antibacterial activity than antibiotic monotherapy. Therefore, numerous articles were published to indicate the synergistic effect of propolis and antibiotics [82,83,84]. For instance, propolis’s ethanolic extract has a synergistic effect (FIC index ≤ 0.5) with aminoglycoside antibiotics (gentamycin, amikacin, and kanamycin), tetracycline, and fusidic acid on the inhibition of *Staphylococcus aureus* growth [61]. *Staphylococcus aureus* MIC values were between 0.128 and 0.512 mg/mL. No antagonistic interaction (FIC index > 4) was identified in this study.

### 7.3. Phenolic Compounds/Polyphenols

The synergistic activities of baicalein, a flavonoid isolated from the root of *Saitellaria baicalensis* *Georgi* with ampicillin or gentamycin against Gram-positive and Gram-negative oral bacteria strains, were studied. Both combinations exhibited synergistic effects (FIC index < 0.375–0.5). *Saitellaria baicalensis Georgi* was determined with MIC values ranging from 0.08 to 0.32 mg/mL against oral bacteria [85]. Another study evaluating the effect between amentoflavone (biflavonoid isolated from *Selaginella tamariscina*) and ampicillin, chloramphenicol, and cefotaxime showed that amentoflavone exhibited a synergistic interaction with antibiotics against the Gram-positive and Gram-negative bacteria studied (FIC index 0.375 to 0.5) except for *Streptococcus mutants*. The results showed that amentoflavone, with a MIC value of 0.004 to 0.032, had remarkable antibacterial activity [86]. In another study, the antimicrobial activity of seven phenolic compounds with six antibiotics against multidrug-resistant bacteria of the ESKAPE group was evaluated. Phenolic compounds on their own revealed little or no inhibitory effects (MIC 0.0125 to 0.4). However, thirty combinations showed antagonistic effects (FIC index > 2) and twenty-four potential synergistic effects (FIC index 1.0 to 1.5) [87]. Recent studies report that plant extracts show different antimicrobial properties against bacterial strains depending on the antibiotic resistance profile [88]. For instance, *Cistus salviifolius* and *Punica granatum* extracts were tested against 100 *Staphylococcus aureus* clinical isolates, which resulted in average MIC values ranging between 0.05 and 0.08 mg/mL. The extract of *Cistus salviifolius* has exerted greater efficacy against strains of *Staphylococcus aureus* resistant to beta-lactam antibiotics and this increased efficacy may be due to the existence of synergy between different classes of polyphenols. However, the extract of *Punica granatum* has shown greater efficacy against strains sensitive to oxacillin and quinolones [89].

### 7.4. Alkaloids

The compound 1-4-naphthoquinone is a natural alkaloid shown to have antibacterial activity against both Gram-positive and Gram-negative bacteria. The MIC values of 1-4-naphthoquinone range from 0.0078 to 0.125 mg/mL. 1,4-naphthoquinone exerts synergy (FIC index ≤ 0.5) with imipenem, cefotaxime, and cefuroxime against methicillin-resistant *Staphylococcus aureus* (MRSA), while against American-type culture collection (ATCC)-cultured MRSA, a synergistic effect was found only between 1,4-naphthoguinone and cefotaxime (FIC index = 0.5). An additive combination with imipenem (FIC index = 1.063) was produced and antagonistic action was identified between 1,4-naphthoquinone and cefuroxime (FIC index = 8.5) [90].

## 8. Mechanisms Underlying the Combination Effects

Synergy in drugs can occur through the following mechanism:

### 8.1. Mechanisms Underlying Synergistic or Antagonistic Antimicrobial Activity

#### 8.1.1. Pharmacodynamic Synergy

Pharmacodynamic synergy results from the targeting of multiple pathways, which may include substrates, enzymes, metabolites, ion channels, ribosomes, and signal cascades [12]. It may also occur through complementary actions, where synergists in a mixture interact with multiple sites of a given pathway and can result in positive regulation of a target or in negative regulation of competing mechanisms [54].

#### 8.1.2. Pharmacokinetic Synergy

Plant-derived compounds can increase the solubility, absorption, transport, distribution or stimulate the metabolism of bioactive constituents. In this way, the bioavailability of compounds is enhanced, resulting in increased efficacy of the extract as compared to individual compounds in isolation [54]. Compounds that improve the solubility of bioactive constituents are a significant type of synergy that is often underestimated. Modulation of compound/drug transport enhances their absorption through disruption of transport barrier, delay of barrier recovery, or reduction of excretion by inhibiting drug effects [91,92]. Modulation of distribution increases the concentration by blocking the compound/drug uptake and inhibiting the metabolic processes that convert a compound/drug into excretable forms. In addition, metabolic modulation stimulates the metabolism of drugs into active forms or inhibits the metabolism of compounds/drugs into inactive forms [54].

#### 8.1.3. Targeting Disease Resistance Mechanisms

The bacterial resistance to beta-lactam antibiotics can be overcome by the combination of beta-lactamase inhibitors with beta-lactam antibiotics. It was reported that a dichloromethane extract of *Vitellaria paradoxa* C.F. Gaertn leaves the activity of ampicillin, oxacillin, and nafcillin synergized against MRSA by targeting PBP2a+/−beta-lactamase enzymes. Oleanolic acid and ursolic acid were found to be the compounds that exerted this synergy [93,94].

#### 8.1.4. Elimination of Adversely Acting Compounds

The elimination or neutralization of adverse effects of a toxic, but bioactive compound by inactive mixture compounds comprises an additional type of synergy. This mechanism does not improve the efficacy of bioactive compounds but rather acts to minimize the adverse effects that an active compound may cause [12].

Although mechanisms by which synergy can occur in drugs are relatively well known, the mechanisms by which medicinal plant-derived compounds exhibit synergetic effects have not yet been fully clarified. Bioactive compounds act in a synergistic or antagonistic manner, and it appears that in most compounds multi-target effects predominate [95]. For example, the following mechanisms of antimicrobial interactions can produce synergy between EOs bioactive compounds: (1) inhibition of several steps in a biochemical pathway; (2) inhibition of enzymes that degrade antimicrobials; (3) interaction of antimicrobials with the cell wall; or (4) interaction with the cell wall resulting in increased uptake of other antimicrobials [96]. Moreover, antagonism is supposed to occur when (1) a combination of bacteriostatic and bactericidal antimicrobials exists; (2) antimicrobials act on the same site; or (3) antimicrobials act with each other [97].

### 8.2. Approaches Identifying Mechanisms of Combination Effects

Determination of the bioactive compounds related to the biological effects of complex mixtures and recognition of the interactions in which they are involved is very important. However, it is also important to identify molecular mechanisms responsible for the combined effects of complex mixtures. This can occur through the following approaches, including targeted biological assays to identify molecules that affect specific molecular targets and evaluation of changes in protein, gene, and metabolic profiles in an untargeted way [98].

#### 8.2.1. Targeted Assays

A common method to evaluate EPI involves the use of an efflux pump substrate that fluoresces when it is contacted with cellular DNA. EPI increases the fluorescence of the substrate because of the increased cellular accumulation. This method was successfully used to discover EPIs from medicinal plant mixtures [99].

#### 8.2.2. Untargeted Approaches

Multi-target effects, whether they are related to a single compound or multiple compounds, can be identified with indirect approaches. A search of medicinal plant compounds using molecular interaction profiles may detect the synergistic mechanisms of action. In addition, the efficacy of medicinal plant mixtures and their effect on molecular targets can be influenced by differences in genes, timing and dosage of therapy, and environment [54].

A visualization approach (“Synergy Maps”) can provide information on the mechanisms of actions by identifying relationships between individual compound characteristics and their combination effects [100]. The use of DNA and RNA microassays is another approach for searching for combination effects within complex mixtures. This approach enables the identification of genes that are regulated by synergistic or antagonistic interactions between the plant species [101]. In silico approaches predicting the mechanisms of action were developed to overcome the time- and material-consuming nature of biological testing. Experimental activity data can be utilized to discover ligand–target relationships and find the biological activities of different molecules [102]. The Functional Signature Ontology (FUSION) maps are used to link natural products to their mechanism of action. Data from measuring gene expression of a representative subset of genes can be combined into FUSION maps to link bioactive molecules to the proteins that they target in cells [103]. Another approach, the network pharmacology approach can predict the interactions between molecules and proteins in a biological system and evaluate the pharmacological effects of natural product mixtures [102].

## 9. Determination of Combination Effects

### 9.1. Collecting Biological Data

The most successful way to collect proper data for understanding combination effects in complex systems is to choose a suitable biological assay for combination testing. Indeed, the establishment of high-quality in vitro testing promises for identifying multi-target compounds in mixtures [103]. Except for carefully collecting the biological assay to study the combined effects, data relevant to the comparison between a compound combination and compounds in isolation should be gathered [11].

Potential combination effects including synergy and antagonism can occur over a wide range of concentrations. Therefore, different ratios of the samples must be tested [11]. Simple assays employing concentration-based methods cannot claim synergy without further in-depth studies because they lack the range of concentration combinations required to evaluate combination effects [104]. Time-based approaches were also applied to identify antimicrobial synergy. These methods involve sampling cultures at regular time intervals and defining synergistic, additive, and antagonistic effects by using a resulting dose–response curve [105].

### 9.2. Assessing Combination Effects

Different reference models that are used to identify the outcome or a given combination may cause confusion concerning the classification of synergistic or antagonistic interactions [28,106]. Several reference models as well as their biological properties are summarized below. 

The combination index (CI) is a practical model used for the quantitative identification of the synergy of multi-compound combination agents acting on the same target/receptor in a fixed ratio. Synergy occurs when the CI value is <1, while additive effect occurs when the CI value is 1 and antagonism exists when the CI value is >1 [107].

The two main reference models are the Bliss independence model [108] and the Loewe additivity model [109]. The Bliss model suggests that each sample has an independent effect, while the Loewe model considers the expected effect as a sample combined with itself. If both models confirm an interaction as synergistic, that interaction should be considered strong synergy. However, if the combination is identified as synergistic by one model only, it should be considered weak synergy [27].

**Figure 2:**
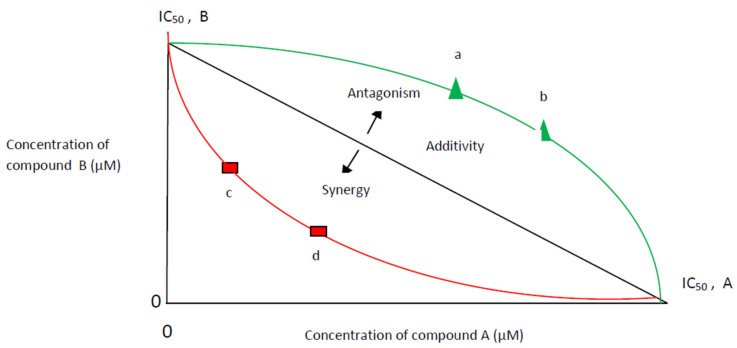
Isobologram model at half maximal inhibitory concentration (IC_50_). Additivity: A line from IC_50_ of compound **A** to IC_50_ of compound **B**; Synergy: any combination points (c,d) lower of additivity line-concave curve; Antagonism: any combination points (a,b) upper of additivity line-convex curve.

The isobole equation, based on the Loewe additivity principle, is widely accepted as one of the most practical models to study combination effects [110]. An isobole or isobologram is the graphical representation of the combined effects of two samples [11,29,111]. The isobologram model is designed to assess the synergistic/antagonistic interactions between two compounds acting on the same target and is less adequate to assess the complex interactions among multiple potential bioactive compounds that may act on a network target [112] (Figure 2).

Another more recently developed model based on both Loewe and Bliss models, the zero-interaction potency model, is related to the assumption that two non-interacting samples cause minimal changes to the dose–response curves. This model potentially identifies the variety of combination effects that occur in different concentration ranges [113].

The systematic analysis/system-to-system (S2S) model was developed to address the multi-target synergistic actions of mixed chemical compounds, with a system of targeted protein/receptors. The S2S studies the multi-target mechanisms of the action of complex compound mixtures and identifies bioactive compounds, which may bind to most of the corresponding targets [114]. It is popular as a valuable tool to assess the synergy of complex medicinal plant formulations [115].

### 9.3. Scoring Biological Data

Most synergy analyses focus on the variations of isobologram and FIC index, which have found many applications [11]. Using measurements of the MIC, the FIC index is calculated according to the formula: FIC_A_ = MIC_A+B_/MIC_A_, FIC_B_ = MIC_B+A_/MIC_B_, FIC index = FIC_A_ + FIC_B_. The MIC_A+B_ value is the MIC of compound A in the presence of compound B, and vice versa [116]. This definition was replaced by a general, widely accepted definition where synergistic interactions are considered to be any values ≤0.5, additive interactions range from 0.5–1.0, non-interactive effects: range from 1.0–4.0, and antagonistic interactions are considered to be any values >4.0 [11,117].

Another score, the delta-score, is visualized using an interaction landscape over all tested dosage combinations to discover any changes in combination effects along with multiple dosages and response levels [113].

The above-described approaches have not yet been applied widely to identify synergy in complex natural products. However, the FIC index is frequently used in natural product research [11].

## 10. Determination of Bioactive Compounds Responsible for Combination Effects

To identify bioactive compounds and to improve the efficacy of medicinal plant extract mixtures, bioactive compounds responsible for the biological identity, whether synergistic, additive, or antagonistic, should be isolated and characterized and their concentrations should be determined. The data that needs to be integrated for the efficient identification of bioactive compound combinations include chemical bioactivity data, gene expression data, targets, and pathway annotations, and gene–protein interaction networks [118]. Metabolomics (the comprehensive analytical approach for the identification and quantification of secondary metabolites in a biological system) is a significant tool for standardization and quality control in medicinal plants [119]. Metabolomics combines sophisticated analytical technologies (mass spectrometry (MS) coupled with various chromatographic separation techniques) with the application of statistical and multi-variant methods for data interpretation. Because of the large number of bioactive compounds and the large variations in abundance, there is no single method available to analyze the whole number of the chemical compounds [120]. 

### 10.1. Methods to Identify Bioactive Molecules

The most commonly used method to identify bioactive compounds is bioassay-guided fractionation. In this method, extracts are separated using different chromatographic techniques, the fractions are evaluated for biological activity and the process is repeated until bioactive compounds are identified and characterized. To avoid the isolation of known bioactive compounds, structural evaluation steps to discard samples containing known bioactive compounds should be taken through high-resolution MS, UV spectroscopy, NMR, and tandem mass spectrometry (MS/MS) molecular networking [121].

### 10.2. Methods to Identify Synergy

The synergy-directed fractionation, a modification of bioassay-guided fractionation, combines chromatographic separation and synergy testing in order to identify synergistic interactions between the bioactive compounds present in a mixture. Through a combination of fractions with a known bioactive compound found in the original extract and testing for combination effects, synergists that did not exhibit activity on their own could be identified [16]. This method uses MS for guided isolation of bioactive compounds, with potential synergistic interactions among extracts that could not have possessed any biological activity through conventional guided fractionation [122].

### 10.3. Metabolomics Methods to Identify Bioactive Compounds

Fractionation approaches focus mainly on the most easily isolated compounds in a mixture rather than those that are active [123]. Therefore, many efforts were made to identify and isolate bioactive compounds by combining the chemical and biological properties of samples under analysis. To achieve this, bioactive compounds were identified based on MS and gas chromatography (GC) and biological data. The combination of MS and GC can be an important analytical tool that separates compounds and identifies their chemical structures [124]. Nuclear magnetic resonance (NMR) spectroscopy is one of the three (the other two being GC–MS and LC–MS) principal analytical methods in metabolomics for profiling and identifying the metabolite in complex mixtures such as plant extracts [125]. NMR-based metabolomics was successfully applied for the identification of antibacterial mechanisms of action of various compounds [126] and also for the characterization of plant secondary metabolites [127]. In particular, the NMR approach coupled with multivariate data analysis identified compounds of medicinal plants contributing to the antiviral activity [128], suggesting actinobacteria and their compounds as a potential control against phytopathogenic bacteria [129] and showing the antimicrobial mechanism of organic acids on *Salmonella enterica* strains [130]. 

Multivariate statistical methods were applied to integrate biological assay data with measurements of chemical compounds, a process that is termed “biochemometrics”. Using more than one statistical model appears to overcome the problems of each model independently [131,132].

The S-plot method is used to plot the correlation and covariance of variables with a given biological activity. In a recent study, this method confirmed differences in metabolite profiles of *Garcinia oblongifolia* (*Clusiacae*) and correlated those differences to differences in biological activity [133]. Another visualization method, the selectivity ratio, showed that bioactive mixture compounds were identified early in the fractionation process, so that isolation attempts by LC coupled to MS/MS concern compounds that were more likely to exert bioactivity [123,134]. Bioactive molecular networking is a process to identify potential bioactive compounds in which nodes connected in a molecular network represent related compounds based on MS/MS fragmentation data, and the size of the nodes corresponds to the predicted bioactivity score [135].

### 10.4. Metabolomics Methods to Identify Synergy

To establish a metabolomic profile, spectroscopic and spectrometric methods are used, such as NMR and MS, and separation methods coupled to mass spectrometric detection, such as high-performance liquid chromatography (HPLC), ultra-HPLC, GC and supercritical fluid chromatography. The selection method is influenced by the matrix and the amount of sample, and the concentration and properties of the metabolites [136]. For example, using LC–MS-based metabolomics, the synergistic activity of a colistin–sulbactam combination was found effective against multidrug-resistant *Acinetobacter baumannii* [137]. 

Flaxomics, a new metabolomics application measures the actual reaction rates (fluxes) of metabolic pathways indirectly by the shifts in metabolic levels. Therefore, the flaxone (total set fluxes) is observing the interactions between all the “-omes”, thus granting a synergistic insight [138]. In a study, the metabolomic profile of *Vibrio alginolyticus* and the role of its metabolism in multi-drug resistance was examined. This was carried out by detecting the metabolic differences of acetyl-CoA fluxes into and through the P-cycle and fatty acid biosynthesis [139].

In a recent study, a large discrepancy between the expected and observed activities of an extract containing the bioactive compounds of berberine and magnolol was noted. The evaluation of this discrepancy indicated the presence of antagonists within the mixture. Using chromatographic separation, the antagonists were separated from bioactive compounds, and the activity of the extract was reinstated [134]. Therefore, predictive methods which can identify bioactive compounds alone may not be capable of determining the complexity of the extract mixture and other methods, which can identify the presence of synergists or antagonists, are required. 

The combination of synergy-directed fractionation with biochemometric analysis could identify synergists and additives in complex medicinal plant-derived extracts. In a study, MS was combined with a biological assay to produce selectivity ratio plots predicting potential synergistic or additive mechanisms between bioactive compounds from *Hydrastis canadensis***,** which enhanced the antimicrobial activity of berberine against *Staphylococcus aureus* [22]. Using this method, bioactive compounds not previously identified with synergy-directed fractionation approaches alone were found as synergists or additives [16]. 

## 11. Challenges of Combination Effects

### 11.1. Biomedical Research and Traditional Uses of Medicinal Plants 

Biomedical research of medicinal plants has focused on the bioactivity of processed medicinal plant products, including mechanisms of medicinal plant parts, isolated fractions, and purified interactive compounds. On the contrary, traditional knowledge-based medicinal plant medicine uses the entire medicinal plant portions. However, there is a tendency to generalize findings from biomedical research on the chemical parts of a medicinal plant to the whole portion of the plant. A challenge is whether biomedical research on processed and possibly enhanced medicinal plant parts or bioactive compounds is applicable to the traditional uses of medicinal plants. If the bioactive compound is extracted from the chemical complex of the medicinal plant, it may not have the same function. Bioactive compounds rarely have the same degree of activity as the unrefined plant-derived extracts at comparable concentrations or doses of the bioactive compounds. This is attributed to the absence of interacting compounds present in the extract [140]. Therefore, reductionist methods of research might have limited applicability to traditional medicinal plant medicine where complex intact plants are used [141].

### 11.2. Models for the Study of Combination Effects

Except for a lack of consensus among the models which are used to define combination effects, there are concerns about how to apply and interpret existing models to analyze combinations [113]. Comparing combination activity to individual activity at the same effect level or dosage is a usual error in studies analyzing synergy. Synergy was reported when the ED50 (median effective dose) value of a combination was significantly lower than that of each individual compound. However, this suggestion cannot distinguish between a synergistic effect and an additive effect as it still compares the effect of the combination with that of individual compounds, which would result in a wrong conclusion. Moreover, these studies were conducted to evaluate the interactions of pharmaceutical drugs and do not take into consideration the complex compound mixtures, effects, and interactions of whole medicinal plant preparations [142].

Considering that the expected outcome of different models, such as the Bliss independence model and the Loewe additivity model, is often dissimilar, it is challenging to conclude from the resulting data. In some instances, combination effects were determined as synergistic by one model but antagonists by another [143]. In addition, each of the models that determine the interactions of bioactive compounds has strengths and limitations, and therefore care needs to be taken when designing a synergistic study of medicinal plant compounds. For example, CI and isobologram models are simple and ideal for studying the interactions of a small number of bioactive compounds with well-defined chemical properties. These models are also designed for single-target treatment and they are of limited use in identifying synergistic effects in combined treatments, especially those in medicinal plant medicine. 

In vivo model systems allow the most comprehensive evaluation of the effects on a living organism [103]. Nonetheless, it is a great challenge to manage the complexity of in vivo systems such as the sacrifice of animal models, the unsuccessful translation from one animal model to another, and the fact that patient-to-patient variability in drug responses is common [26]. To overcome some of these challenges, researchers work with relevant cellular systems to identify combination effects in vitro [144] and challenges of relating in vitro findings to in vivo and clinical practice are raised by some authors [145]. However, the biological assay sample may be subjected to a chemical change due to the environment, making interpretation of the results a real concern [146]. Moreover, the chemical complexity and variability of medicinal plant compounds and the conflicting information on their mechanisms of action further increase the challenges for the design conduct and interpretation of natural product clinical trials [147].

### 11.3. Determination of Bioactive Compounds and Synergy

Identifying multiple bioactive molecules that may contribute in a synergistic, additive, or antagonistic manner to biological activity represents a critical challenge in the study of medicinal plant extracts. Another challenge with extracts is that their greater complexity is more likely to be associated with all types of interaction [148].

An added challenge is the need to determine the bioactive compounds responsible for the antimicrobial activity in complex mixtures and their possible interactions. In fact, it is difficult to apply bioassay-guided fractionation when the activity of the mixture is due to hundreds or thousands of bioactive compounds which are often unknown and may have low potency [15]. Despite these challenges, studying only one or two bioactive compounds in an extract is not reasonable. Almost complete identification of main compounds can sometimes be possible for some medicinal plants. However, in other cases, there is the possibility that certain bioactive compounds may not be isolated [15]. Several explanations were proposed for this which may be summarized as follows: (1) the quality of ethnopharmacological studies is poor; (2) preclinical laboratory methodologies are often different from local practices; (3) a whole mixture is necessary for the therapeutic effect if synergy exists or suspected; (4) whole plant material containing some specific bioactive compounds may protect other compounds from decomposition; (5) bioactive compounds may not have been completely identified, although some of the chemistry is known, due to inadequate plant material processing or fractionation approach; (6) degradation of bioactive compounds during fractionation may happen; and (7) poor biological models to demonstrate activities may be chosen. 

When addressing the challenge of conducting bioassay-guided fractionation to decide which mixture compounds to target for isolation, it is important to focus on the most bioactive fraction from a crude extract [149,150]. However, the concentration of bioactive compounds may be low following fractionation [110] or the biological activity of the extract may be progressively lost through the fractionation but the reason for that is hard to explain [151]. Additionally, using the chromatographic profile of active and inactive fractions, one may be biased by the tallest peaks of those most clearly separated from other peaks, although these may correspond neither to the most abundant nor to the most active compounds. The extraction of proper information and its adequate interpretation from the vast amount of data is challenging in metabolomics. The application of advanced statistical and multi-variant analysis tools has shown that changes are needed to deal with large data sets [152].

### 11.4. Biochemometrics to Target Bioactive Compounds

With the emergence of new methods, such as metabolomics, proteomics and transcriptomics, several strategies are currently used to highlight bioactive compounds from medicinal plant complex mixtures at a very early stage [153]. Such methods would overcome some bioassay-guided fractionation limitations, and specifically study bioactive compounds responsible for the activity. However, most of these methods use only one statistical model for interpretation which is not well adapted to elucidate chemical profile and bioactivity relationships. Therefore, a combination of statistical models may increase the performance of biochemometrics. The authors of a study proposed a comprehensive workflow that combined explorative-solid phase extraction and biochemometrics analysis with the integration of four statistical models [154].

Using biochemometrics methods, chemical and biological data can be interpreted when multivariate statistics and potential bioactive compounds identified early in the fractionation process are used [124,134]. These methods, however, do not provide basic information about potential unknown bioactive compounds, preventing the ability to optimize isolation efforts. To overcome this challenge, a combination of selectivity ratio analysis and molecular networking workflow within the Global Natural Product Search was used to identify putative bioactive compounds not previously known to exhibit antimicrobial activity [143]. Moreover, by considering both MS/MS fragmentation data and area data, molecular networking provides the re-isolation, compound annotation, and identification of potentially active compounds in one step [135,155].

## 12. Study Limitation

As was mentioned before, identifying which bioactive compounds in complex mixtures exhibit biological effects can be challenging. To overcome this limitation, pulsed ultrafiltration MS was used for the screening of mixtures such as medicinal plant extracts. Moreover, these extracts may not be completely safe due to the diversity of bioactive compounds in them, when used as antimicrobial agents and, therefore, a need to analyze the bioactive compounds more clearly and identify the formation of residues is required [156]. 

A limitation of bioassay-guided fractionation is that fractions throughout the isolation process may be at very low concentration to present biological effects and therefore be overlooked. It is also considered a time-consuming, risky, and costly method [157,158]. Synergy-directed fractionation would facilitate the identification of many bioactive compounds including synergists. A limitation of synergy-directed fractionation is that this method is biased toward the bioactive compounds that are easily isolated. Although this method focuses on the most bioactive fractions, it is impossible to identify all the bioactive compounds that they contain due to the complexity of fractions. Therefore, the identified bioactive compounds may represent only a part of these responsible for the activity of the complex mixture [65].

Another important limitation is that in vitro assay data to assess bioactive compound activity may not necessarily translate to in vivo activity, partially due to the complexity of biological systems and the effects of biological agents that are not identified in vitro models. It is significant to know that the extent of the utility of any bioassay-guided fractionation method is limited by the availability of a translatable biological assay [158]. Moreover, synergistic effects demonstrated in many studies do not necessarily reflect clinical therapeutic superiority. The clinical benefits of complex active compound combinations must be confirmed in meticulous clinical trials.

Reference and linear regression models are subject to limitations [159]. For example, a limitation of the FIC index is the focus on a single parameter. Despite this limitation, the FIC index and isobologram analysis have found the widest application in medicinal plant research [11]. Similarly, the linear regression models used to predict bioactive compounds are limited because true linear relationships do not often exist, particularly when evaluating mixtures with multiple unknown combination effects [26].

## 13. Future Directions

The development of current and new technologies will result in the identification of plant-derived antimicrobials and their synergy with antibiotics [160,161,162,163], as well as the clarification of their complex mechanisms of interactions [164]. Therefore, the use of modern methods, antimicrobial testing with standardized protocols, and quality controls of plant materials are necessary. For example, studying mechanisms using molecular biology methods for the identified plant-derived bioactive compounds individually in combination may possibly facilitate the development of more efficient new antimicrobial agents [165]. Further studies on the complete analysis of the molecular interaction profiles of the bioactive compounds are needed to provide a more detailed picture of their antimicrobial activity. 

Research of plant-derived bioactive compound synergy can be further facilitated by new knowledge of their synergistic interactions. Studies on untargeted methods to identify molecular targets of synergy and unknown synergistic or antagonistic mechanisms of action will be of great importance. Integrated methods completing tasks such as the identification of bioactive mixture compounds [165], the determination of the nature of their interactions, and the classification of their mechanisms of action simultaneously should be developed. Moreover, biomarkers derived from bioactive compounds that regulate synergistic interactions may be essential for defining treatment strategies. 

More research should be focused on the structural clarification of medicinal plant extracts to identify and isolate new natural antimicrobials and elucidate the mechanisms of action of their combinational effects [166]. Additionally, new insights are needed in the research of models investigating systematic connections and whole system responses over time which should prevail over models that reduce medicinal plant medicine to parts [155]. New workflows based on the combination of both extraction protocols and biochemometrics could be a perspective in the context of potentially bioactive compound discovery screening programs.

## 14. Conclusions

Unquestionable definitions of synergy and antagonism remain deceptive and a wider consensus on the terminology used for interaction evaluation is required to standardize future research actions. Moreover, there is no definitive consensus about which reference models are best for defining combination effects. Despite this, isobologram analysis and the FIC index have found the widest use in natural product research. 

Approaches for medicinal plant-based antimicrobial synergy research are developing but in vitro research is not progressing into clinical studies. Indeed, the process of selecting complex medicinal plant bioactive compounds for in vitro, in vivo, and clinical studies is challenging. Therefore, the design of meticulous and reproducible studies is obligatory to prevent unnecessary effort for studies where natural product selection was not carried out with strict criteria. 

The use of biochemometrics to combine bioassay data with chromatographic or spectrometric metabolite profiles can lead the isolation process toward bioactive compounds that may exhibit relevant biological activity.

Synergy in medicinal plant compounds is significant in the context of antimicrobial resistance, not just because of synergists and antimicrobials, but because it adds to the systemic ways of knowing about its diversity, adaptability, and complexities. Progress in synergy research will rationalize the therapeutic effects of the complex mixtures of plant bioactive compounds and enhance the possibility of using new antimicrobials. 

Classifying combination effects within complex mixtures remains a challenge, particularly when the majority of established methods are used to reduce the complexity of mixtures. It is time to examine bioactive compound interactions and not to use the reductionist method to identify a single bioactive compound in medicinal plant research. The development of new combination therapies will facilitate the study of bioactive compound mixtures that naturally exist in medicinal plants. 

Only through understanding the nature of interactions between medicinal plant-derived bioactive compounds will we be able to create safe and efficient preparations for the treatment of infectious diseases. However, many challenges and unknown factors, especially when evaluating the process, analysis and function of bioactive compounds, must be overcome and solved.

## Figures and Tables

**Table 1:** Combination effects of propolis’s flavonoids against nine pathogens. Reprinted with permission from Ref. [63]. Permission to use the data has been obtained from Elsevier, 2019.

Pathogen	Pinocembrin-Chrysin		Pinocembrin-Galangin		Chrysin-Galangin	
	FIC Index *	Interaction	FIC Index	Interaction	FIC Index	Interaction
Gram (+) bacteria						
*S. aureus*	0.57	Additive	1.10	Non-interactive	0.75	Additive
*L. monocytogenes*	0.76	Additive	0.19	Synergistic	0.26	Synergistic
*E. faecalis*	1.00	Non-interactive	1.00	Non-interactive	0.81	Additive
Gram (−) bacteria						
*E. coli*	1.12	Non-interactive	0.63	Additive	0.65	Additive
*K. pneumonia*	1.00	Non-interactive	1.00	Additive	1.00	Non-interactive
*P. aeruginosa*	0.82	Addittive	0.52	Non-interactive	0.49	Synergistic
Fungal pathogens						
*C. albicans*	1.23	Non-interactive	0.74	Additive	1.00	Non-interactive
*C. neoformans*	1.00	Non-interactive	0.40	Synergistic	1.00	Non-interactive
*C. tropicalis*	1.13	Non-interactive	0.44	Synergistic	0.14	Synergistic

* FIC index: Fractional inhibitory concentration index.

## Data Availability

Not applicable.

## References

[1] Applequist W.L., Miller J.S. (2013). Selection and Authentication of Botanical Materials for the Development of Analytical Methods. Anal. Bioanal. Chem..

[2] Sorkin B.C., Kuszak A.J., Williamson J.S., Hopp D.C., Betz J.M. (2016). The Challenge of Reproducibility and Accuracy in Nutrition Research: Resources and Pitfalls. Adv. Nutr..

[3] Simmler C., Graham J.G., Chen S.-N., Pauli G.F. (2018). Integrated Analytical Assets Aid Botanical Authenticity and Adulteration Management. Fitoterapia.

[4] Fenclova M., Novakova A., Viktorova J., Jonatova P., Dzuman Z., Ruml T., Kren V., Hajslova J., Vitek L., Stranska-Zachariasova M. (2019). Poor Chemical and Microbiological Quality of the Commercial Milk Thistle-Based Dietary Supplements May Account for Their Reported Unsatisfactory and Non-Reproducible Clinical Outcomes. Sci. Rep..

[5] Wang J., Hodes G.E., Zhang H., Zhang S., Zhao W., Golden S.A., Bi W., Menard C., Kana V., Leboeuf M. (2018). Epigenetic Modulation of Inflammation and Synaptic Plasticity Promotes Resilience against Stress in Mice. Nat. Commun..

[6] Boudreau A., Poulev A., Ribnicky D.M., Raskin I., Rathinasabapathy T., Richard A.J., Stephens J.M. (2019). Distinct Fractions of an Artemisia Scoparia Extract Contain Compounds with Novel Adipogenic Bioactivity. Front. Nutr..

[7] Munyangi J., Cornet-Vernet L., Idumbo M., Lu C., Lutgen P., Perronne C., Ngombe N., Bianga J., Mupenda B., Lalukala P. (2019). Artemisia Annua and Artemisia Afra Tea Infusions vs. Artesunate-Amodiaquine (ASAQ) in Treating Plasmodium Falciparum Malaria in a Large Scale, Double Blind, Randomized Clinical Trial. Phytomedicine.

[8] Hopp D.C. Past and Future Research at National Center for Complementary and Integrative Health with Respect to Botanicals—American Botanical Council. https://www.herbalgram.org/resources/herbalgram/issues/107/table-of-contents/hg107-feat-nccih/.

[9] Newman D.J., Cragg G.M. (2020). Natural Products as Sources of New Drugs over the Nearly Four Decades from 01/1981 to 09/2019. J. Nat. Prod..

[10] Efferth T., Koch E. (2011). Complex Interactions between Phytochemicals. The Multi-Target Therapeutic Concept of Phytotherapy. Curr. Drug Targets.

[11] van Vuuren S., Viljoen A. (2011). Plant-Based Antimicrobial Studies—Methods and Approaches to Study the Interaction between Natural Products. Planta Med..

[12] Wagner H., Ulrich-Merzenich G. (2009). Synergy Research: Approaching a New Generation of Phytopharmaceuticals. Phytomedicine.

[13] Burfield T., Reekie S.-L. (2005). Mosquitoes, Malaria and Essential Oils. Int. J. Aromather..

[14] Raskin I., Ripoll C. (2004). Can an Apple a Day Keep the Doctor Away?. Curr. Pharm. Des..

[15] Enke C.G., Nagels L.J. (2011). Undetected Components in Natural Mixtures: How Many? What Concentrations? Do They Account for Chemical Noise? What Is Needed to Detect Them?. Anal. Chem..

[16] Junio H.A., Sy-Cordero A.A., Ettefagh K.A., Burns J.T., Micko K.T., Graf T.N., Richter S.J., Cannon R.E., Oberlies N.H., Cech N.B. (2011). Synergy Directed Fractionation of Botanical Medicines: A Case Study with Goldenseal (*Hydrastis Canadensis*). J. Nat. Prod..

[17] Stermitz F.R., Lorenz P., Tawara J.N., Zenewicz L.A., Lewis K. (2000). Synergy in a Medicinal Plant: Antimicrobial Action of Berberine Potentiated by 5′-Methoxyhydnocarpin, a Multidrug Pump Inhibitor. Proc. Natl. Acad. Sci. USA.

[18] Stermitz F.R., Scriven L.N., Tegos G., Lewis K. (2002). Two Flavonols from Artemisa Annua Which Potentiate the Activity of Berberine and Norfloxacin against a Resistant Strain of Staphylococcus Aureus. Planta Med..

[19] Ulrich-Merzenich G., Panek D., Zeitler H., Vetter H., Wagner H. (2010). Drug Development from Natural Products: Exploiting Synergistic Effects. Indian J. Exp. Biol..

[20] Abreu A.C., McBain A.J., Simões M. (2012). Plants as Sources of New Antimicrobials and Resistance-Modifying Agents. Nat. Prod. Rep..

[21] Eloff J.N. (2004). Quantification the Bioactivity of Plant Extracts during Screening and Bioassay Guided Fractionation. Phytomedicine.

[22] Britton E.R., Kellogg J.J., Kvalheim O.M., Cech N.B. (2018). Biochemometrics to Identify Synergists and Additives from Botanical Medicines: A Case Study with Hydrastis Canadensis (Goldenseal). J. Nat. Prod..

[23] Bunterngsook B., Eurwilaichitr L., Thamchaipenet A., Champreda V. (2015). Binding Characteristics and Synergistic Effects of Bacterial Expansins on Cellulosic and Hemicellulosic Substrates. Bioresour. Technol..

[24] Chevereau G., Bollenbach T. (2015). Systematic Discovery of Drug Interaction Mechanisms. Mol. Syst. Biol..

[25] Piggott J.J., Townsend C.R., Matthaei C.D. (2015). Reconceptualizing Synergism and Antagonism among Multiple Stressors. Ecol. Evol..

[26] Pemovska T., Bigenzahn J.W., Superti-Furga G. (2018). Recent Advances in Combinatorial Drug Screening and Synergy Scoring. Curr. Opin. Pharmacol..

[27] Tang J., Wennerberg K., Aittokallio T. (2015). What Is Synergy? The Saariselkä Agreement Revisited. Front. Pharmacol..

[28] Berenbaum M.C. (1989). What Is Synergy?. Pharmacol. Rev..

[29] Rather M.A., Bhat B.A., Qurishi M.A. (2013). Multicomponent Phytotherapeutic Approach Gaining Momentum: Is the “One Drug to Fit All” Model Breaking Down?. Phytomedicine.

[30] Williamson–2001–Phytomedicine International Journal of Phytotherapy and Phytopharmacology|PDF|Herbalism|Tetrahydrocannabinol. Scribd. https://www.scribd.com/document/372080329/Williamson-2001-Phytomedicine-International-Journal-of-Phytotherapy-and-Phytopharmacology.

[31] Jia J., Zhu F., Ma X., Cao Z.W., Li Y.X., Chen Y.Z. (2009). Mechanisms of Drug Combinations: Interaction and Network Perspectives. Nat. Rev. Drug Discov..

[32] Siegenfeld A.F., Bar-Yam Y. (2020). An Introduction to Complex Systems Science and Its Applications. Complexity.

[33] Zimmerman B., Lindberg C., Plsek P. (2008). Edgeware: Lessons from Complexity Science for Health Care Leaders.

[34] Spelman K., Duke J.A., Bogenschutz-Godwin M.J. (2006). The Synergy Principle in Plants, Pathogens, Insects, Herbivores and Humans. Natural Products from Plants.

[35] Nakabayashi R., Saito K. (2013). Metabolomics for Unknown Plant Metabolites. Anal. Bioanal. Chem..

[36] Micozzi M.S. (2011). Fundamentals of Complementary and Alternative Medicine.

[37] Ganora L. (2009). Herbal Constituents: Foundations of Phytochemistry.

[38] Koithan M., Bell I.R., Niemeyer K., Pincus D. (2012). A Complex Systems Science Perspective for Whole Systems of Complementary and Alternative Medicine Research. Komplementmed.

[39] Bell I.R., Koithan M., Brooks A.J. (2013). Testing the Nanoparticle-Allostatic Cross Adaptation-Sensitization Model for Homeopathic Remedy Effects. Homeopathy.

[40] Niemeyer K., Bell I.R., Koithan M. (2013). Traditional Knowledge of Western Herbal Medicine and Complex Systems Science. J. Herb. Med..

[41] Hemaiswarya S., Kruthiventi A.K., Doble M. (2008). Synergism between Natural Products and Antibiotics against Infectious Diseases. Phytomedicine.

[42] Datta S., Kumar Pal N.K., Nandy A.K. (2011). Inhibition of the Emergence of Multi Drug Resistant Staphylococcus Aureus by Withania Somnifera Root Extracts. Asian Pac. J. Trop. Med..

[43] Buhner S.H. (2002). The Lost Language of Plants: The Ecological Importance of Plant Medicine to Life on Earth.

[44] Bone K., Mills S. (2013). Principles and Practice of Phytotherapy.

[45] Kourtesi C., Ball A.R., Huang Y.-Y., Jachak S.M., Vera D.M.A., Khondkar P., Gibbons S., Hamblin M.R., Tegos G.P. (2013). Microbial Efflux Systems and Inhibitors: Approaches to Drug Discovery and the Challenge of Clinical Implementation. Open Microbiol. J..

[46] Alviano D.S., Alviano C.S. (2009). Plant Extracts: Search for New Alternatives to Treat Microbial Diseases. Curr. Pharm. Biotechnol..

[47] Palaniappan K., Holley R.A. (2010). Use of Natural Antimicrobials to Increase Antibiotic Susceptibility of Drug Resistant Bacteria. Int. J. Food Microbiol..

[48] Williamson E.M. (2001). Synergy and Other Interactions in Phytomedicines. Phytomedicine.

[49] Beckstrom-Sternberg S.M., Duke J.A., Charalambous G. (1994). Potential for Synergistic Action of Phytochemicals in Spices. Spices Herbs and Edible Fungi.

[50] Djouahri A., Saka B., Boudarene L., Benseradj F., Aberrane S., Aitmoussa S., Chelghoum C., Lamari L., Sabaou N., Baaliouamer A. (2014). In Vitro Synergistic/Antagonistic Antibacterial and Anti-Inflammatory Effect of Various Extracts/Essential Oil from Cones of Tetraclinis Articulata (Vahl) Masters with Antibiotic and Anti-Inflammatory Agents. Ind. Crops Prod..

[51] Bush K., Courvalin P., Dantas G., Davies J., Eisenstein B., Huovinen P., Jacoby G.A., Kishony R., Kreiswirth B.N., Kutter E. (2011). Tackling Antibiotic Resistance. Nat. Rev. Microbiol..

[52] Eliopoulos G.M., Eliopoulos C.T. (1988). Antibiotic Combinations: Should They Be Tested?. Clin. Microbiol. Rev..

[53] Rasoanaivo P., Wright C.W., Willcox M.L., Gilbert B. (2011). Whole Plant Extracts versus Single Compounds for the Treatment of Malaria: Synergy and Positive Interactions. Malar. J..

[54] Ma X.H., Zheng C.J., Han L.Y., Xie B., Jia J., Cao Z.W., Li Y.X., Chen Y.Z. (2009). Synergistic Therapeutic Actions of Herbal Ingredients and Their Mechanisms from Molecular Interaction and Network Perspectives. Drug Discov. Today.

[55] Langeveld W.T., Veldhuizen E.J.A., Burt S.A. (2014). Synergy between Essential Oil Components and Antibiotics: A Review. Crit. Rev. Microbiol..

[56] Bassolé I.H.N., Lamien-Meda A., Bayala B., Tirogo S., Franz C., Novak J., Nebié R.C., Dicko M.H. (2010). Composition and Antimicrobial Activities of Lippia Multiflora Moldenke, Mentha x Piperita, L. and *Ocimum Basilicum*, L. Essential Oils and Their Major Monoterpene Alcohols Alone and in Combination. Molecules.

[57] Pei R.-S., Zhou F., Ji B.-P., Xu J. (2009). Evaluation of Combined Antibacterial Effects of Eugenol, Cinnamaldehyde, Thymol, and Carvacrol against *E. Coli* with an Improved Method. J. Food Sci..

[58] Stević T., Berić T., Šavikin K., Soković M., Gođevac D., Dimkić I., Stanković S. (2014). Antifungal Activity of Selected Essential Oils against Fungi Isolated from Medicinal Plant. Ind. Crops Prod..

[59] Hossain F., Follett P., Dang Vu K., Harich M., Salmieri S., Lacroix M. (2016). Evidence for Synergistic Activity of Plant-Derived Essential Oils against Fungal Pathogens of Food. Food Microbiol..

[60] Mutlu-Ingok A., Devecioglu D., Dikmetas D.N., Karbancioglu-Guler F., Capanoglu E. (2020). Antibacterial, Antifungal, Antimycotoxigenic, and Antioxidant Activities of Essential Oils: An Updated Review. Molecules.

[61] Grecka K., Kuś P.M., Okińczyc P., Worobo R.W., Walkusz J., Szweda P. (2019). The Anti-Staphylococcal Potential of Ethanolic Polish Propolis Extracts. Molecules.

[62] Inui S., Hatano A., Yoshino M., Hosoya T., Shimamura Y., Masuda S., Ahn M.-R., Tazawa S., Araki Y., Kumazawa S. (2014). Identification of the Phenolic Compounds Contributing to Antibacterial Activity in Ethanol Extracts of Brazilian Red Propolis. Nat. Prod. Res..

[63] Kharsany K., Viljoen A., Leonard C., Vuuren S.V. (2019). van. The New Buzz: Investigating the Antimicrobial Interactions between Bioactive Compounds Found in South African Propolis. J. Ethnopharmacol..

[64] Luján M.D.R.M., Reséndez A.M., Barrón G.S.G., Carrillo J.L.R., Inungaray M.L.C. (2018). Antibacterial Activity and Phenolic Content of Propolis Extracts Obtained by Different Extraction Methods. Nova Sci..

[65] Leyte-Lugo M., Britton E.R., Foil D.H., Brown A.R., Todd D.A., Rivera-Chávez J., Oberlies N.H., Cech N.B. (2017). Secondary Metabolites from the Leaves of the Medicinal Plant Goldenseal (*Hydrastis Canadensis*). Phytochem. Lett..

[66] Ettefagh K.A., Burns J.T., Junio H.A., Kaatz G.W., Cech N.B. (2011). Goldenseal (*Hydrastis Canadensis*, L.) Extracts Synergistically Enhance the Antibacterial Activity of Berberine via Efflux Pump Inhibition. Planta Med..

[67] Wang L., Zhang S.-Y., Chen L., Huang X.-J., Zhang Q.-W., Jiang R.-W., Yao F., Ye W.-C. (2014). New Enantiomeric Isoquinoline Alkaloids from Coptis Chinensis. Phytochem. Lett..

[68] White N.J. (2008). Qinghaosu (Artemisinin): The Price of Success. Science.

[69] Suberu J.O., Gorka A.P., Jacobs L., Roepe P.D., Sullivan N., Barker G.C., Lapkin A.A. (2013). Anti-Plasmodial Polyvalent Interactions in Artemisia Annua, L. Aqueous Extract–Possible Synergistic and Resistance Mechanisms. PLoS ONE.

[70] Elford B.C., Roberts M.F., Phillipson J.D., Wilson R.J. (1987). Potentiation of the Antimalarial Activity of Qinghaosu by Methoxylated Flavones. Trans. R. Soc. Trop. Med. Hyg..

[71] Liu K.C., Yang S.L., Roberts M.F., Elford B.C., Phillipson J.D. (1992). Antimalarial Activity of Artemisia Annua Flavonoids from Whole Plants and Cell Cultures. Plant Cell Rep..

[72] Yang S.K., Yap P.S.X., Krishnan T., Yusoff K., Chan K.G., Yap W.S., Lai K.S., Lim S.H.E. (2018). Mode of Action: Synergistic Interaction of Peppermint (Mentha x Piperita, L. Carl) Essential Oil and Meropenem against Plasmid-Mediated Resistant, *E. Coli*. Rec. Nat. Prod..

[73] Moussaoui F., Alaoui T. (2016). Evaluation of Antibacterial Activity and Synergistic Effect between Antibiotic and the Essential Oils of Some Medicinal Plants. Asian Pac. J. Trop. Biomed..

[74] Nafis A., Kasrati A., Jamali C.A., Custódio L., Vitalini S., Iriti M., Hassani L. (2020). A Comparative Study of the in Vitro Antimicrobial and Synergistic Effect of Essential Oils from *Laurus Nobilis*, L. and *Prunus Armeniaca*, L. from Morocco with Antimicrobial Drugs: New Approach for Health Promoting Products. Antibiotics.

[75] Knezevic P., Aleksic V., Simin N., Svircev E., Petrovic A., Mimica-Dukic N. (2016). Antimicrobial Activity of Eucalyptus Camaldulensis Essential Oils and Their Interactions with Conventional Antimicrobial Agents against Multi-Drug Resistant Acinetobacter Baumannii. J. Ethnopharmacol..

[76] Zhang D., Hu H., Rao Q., Zhao Z. (2011). Synergistic Effects and Physiological Responses of Selected Bacterial Isolates from Animal Feed to Four Natural Antimicrobials and Two Antibiotics. Foodborne Pathog. Dis..

[77] Moon S.-E., Kim H.-Y., Cha J.-D. (2011). Synergistic Effect between Clove Oil and Its Major Compounds and Antibiotics against Oral Bacteria. Arch. Oral. Biol..

[78] Ilić B.S., Miladinović D.L., Kocić B.D., Spalović B.R., Marković M.S., Čolović H., Nikolić D.M. (2017). Chemoinformatic Investigation of Antibiotic Antagonism: The Interference of Thymus Glabrescens Essential Oil Components with the Action of Streptomycin. Nat. Prod. Commun..

[79] Salvagno L., Sblano S., Fracchiolla G., Corbo F., Clodoveo M., Rosato A. (2020). Antibiotics—Mentha Piperita Essential Oil Synergism Inhibits Mature Bacterial Biofilm. Chem. Today.

[80] Rosato A., Sblano S., Salvagno L., Carocci A., Clodoveo M.L., Corbo F., Fracchiolla G. (2020). Anti-Biofilm Inhibitory Synergistic Effects of Combinations of Essential Oils and Antibiotics. Antibiotics.

[81] Huang S., Zhang C.P., Wang K., Li G.Q., Hu F.L. (2014). Recent advances in the chemical composition of propolis. Molecules..

[82] Wojtyczka R.D., Dziedzic A., Idzik D., Kępa M., Kubina R., Kabała-Dzik A., Smoleń-Dzirba J., Stojko J., Sajewicz M., Wąsik T.J. (2013). Susceptibility of Staphylococcus Aureus Clinical Isolates to Propolis Extract Alone or in Combination with Antimicrobial Drugs. Molecules.

[83] Ong T.H., Chitra E., Ramamurthy S., Ling C.C.S., Ambu S.P., Davamani F. (2019). Cationic Chitosan-Propolis Nanoparticles Alter the Zeta Potential of S. Epidermidis, Inhibit Biofilm Formation by Modulating Gene Expression and Exhibit Synergism with Antibiotics. PLoS ONE.

[84] Przybyłek I., Karpiński T.M. (2019). Antibacterial Properties of Propolis. Molecules.

[85] Jang E.-J., Cha S.-M., Choi S.-M., Cha J.-D. (2014). Combination Effects of Baicalein with Antibiotics against Oral Pathogens. Arch. Oral. Biol..

[86] Hwang J.H., Choi H., Woo E.-R., Lee D.G. (2013). Antibacterial Effect of Amentoflavone and Its Synergistic Effect with Antibiotics. J. Microbiol. Biotechnol..

[87] Buchmann D., Schultze N., Borchardt J., Böttcher I., Schaufler K., Guenther S. (2022). Synergistic Antimicrobial Activities of Epigallocatechin Gallate, Myricetin, Daidzein, Gallic Acid, Epicatechin, 3-Hydroxy-6-Methoxyflavone and Genistein Combined with Antibiotics against ESKAPE Pathogens. J. Appl. Microbiol..

[88] Atef N.M., Shanab S.M., Negm S.I., Abbas Y.A. (2019). Evaluation of Antimicrobial Activity of Some Plant Extracts against Antibiotic Susceptible and Resistant Bacterial Strains Causing Wound Infection. Bull. Nat. Res. Cent..

[89] Álvarez-Martínez F.J., Rodríguez J.C., Borrás-Rocher F., Barrajón-Catalán E., Micol V. (2021). The Antimicrobial Capacity of Cistus Salviifolius and Punica Granatum Plant Extracts against Clinical Pathogens Is Related to Their Polyphenolic Composition. Sci Rep.

[90] Yap J.K.Y., Tan S.Y.Y., Tang S.Q., Thien V.K., Chan E.W.L. (2021). Synergistic Antibacterial Activity Between 1,4-Naphthoquinone and β-Lactam Antibiotics Against Methicillin-Resistant Staphylococcus Aureus. Microb. Drug Resist..

[91] Stavri M., Piddock L.J.V., Gibbons S. (2007). Bacterial efflux pump inhibitors from natural sources. J. Antimicrob. Chemother..

[92] Adams M., Mahringer A., Kunert O., Fricker G., Efferth T., Bauer R. (2008). Cytotoxicity and P-Glycoprotein Modulating Effects of Quinolones and Indoloquinazolines from the Chinese Herb Evodia Rutaecarpa. Planta. Medica..

[93] Bush K., Jacoby G.A. (2010). Updated Functional Classification of Beta-Lactamases. Antimicrob. Agents Chemother..

[94] Catteau L., Olson J., Van Bambeke F., Leclercq J., Nizet V. (2017). Ursolic Acid from Shea Butter Tree (*Vitellaria Paradoxa*) Leaf Extract Synergizes with β-Lactams against Methicillin-Resistant Staphylococcus Aureus. FASEB J..

[95] Wagner H. (2005). Natural Products Chemistry and Phytomedicine in the 21st Century: New Developments and Challenges. Pure and Applied Chemistry. Pure Appl. Chem..

[96] Pillai S.K., Moellering R.C., Eliopoulos G.M., Lorian V. (2005). Antimicrobial Combinations. Antibiotics in Laboratory Medicine.

[97] Roller S. (2003). Natural Antimicrobials for the Minimal Processing of Foods.

[98] Chen S., Jiang H., Cao Y., Wang Y., Hu Z., Zhu Z., Chai Y. (2016). Drug Target Identification Using Network Analysis: Taking Active Components in Sini Decoction as an Example. Sci. Rep..

[99] Brown A.R., Ettefagh K.A., Todd D., Cole P.S., Egan J.M., Foil D.H., Graf T.N., Schindler B.D., Kaatz G.W., Cech N.B. (2015). A Mass Spectrometry-Based Assay for Improved Quantitative Measurements of Efflux Pump Inhibition. PLoS ONE.

[100] Lewis R., Guha R., Korcsmaros T., Bender A. (2015). Synergy Maps: Exploring Compound Combinations Using Network-Based Visualization. J. Cheminform..

[101] Panossian A., Seo E.-J., Wikman G., Efferth T. (2015). Synergy Assessment of Fixed Combinations of Herba Andrographidis and Radix Eleutherococci Extracts by Transcriptome-Wide Microarray Profiling. Phytomedicine.

[102] Xiong Y., Hu Y., Chen L., Zhang Z., Zhang Y., Niu M., Cui X. (2019). Unveiling Active Constituents and Potential Targets Related to the Hematinic Effect of Steamed Panax Notoginseng Using Network Pharmacology Coupled with Multivariate Data Analyses. Front. Pharmacol..

[103] Potts M.B., Kim H.S., Fisher K.W., Hu Y., Carrasco Y.P., Bulut G.B., Ou Y.-H., Herrera-Herrera M.L., Cubillos F., Mendiratta S. (2013). Using Functional Signature Ontology (FUSION) to Identify Mechanisms of Action for Natural Products. Sci. Signal..

[104] Ocana A., Amir E., Yeung C., Seruga B., Tannock I.F. (2012). How Valid Are Claims for Synergy in Published Clinical Studies?. Ann. Oncol..

[105] Tam V.H., Schilling A.N., Nikolaou M. (2005). Modelling Time-Kill Studies to Discern the Pharmacodynamics of Meropenem. J. Antimicrob. Chemother..

[106] Lee S. (2010). Drug Interaction: Focusing on Response Surface Models. Korean J. Anesthesiol..

[107] Chou T.-C. (2010). Drug Combination Studies and Their Synergy Quantification Using the Chou-Talalay Method. Cancer Res..

[108] Bliss C.I. (1939). The Toxicity of Poisons Applied Jointly 1. Ann. Appl. Biol..

[109] Loewe S. (1953). The Problem of Synergism and Antagonism of Combined Drugs. Arzneimittelforschung.

[110] Heinrich M., Williamson E.M., Gibbons S., Barnes J., Prieto-Garcia J. (2017). Fundamentals of Pharmacognosy and Phytotherapy E-Book.

[111] Lederer S., Dijkstra T.M.H., Heskes T. (2018). Additive Dose Response Models: Explicit Formulation and the Loewe Additivity Consistency Condition. Front. Pharmacol..

[112] Gu P., Chen H. (2014). Modern Bioinformatics Meets Traditional Chinese Medicine. Brief. Bioinform..

[113] Yadav B., Wennerberg K., Aittokallio T., Tang J. (2015). Searching for Drug Synergy in Complex Dose-Response Landscapes Using an Interaction Potency Model. Comput. Struct. Biotechnol. J..

[114] Wang X., Xu X., Tao W., Li Y., Wang Y., Yang L. (2012). A Systems Biology Approach to Uncovering Pharmacological Synergy in Herbal Medicines with Applications to Cardiovascular Disease. Evid. Based Complement. Altern. Med..

[115] Lee S. (2015). Systems Biology—A Pivotal Research Methodology for Understanding the Mechanisms of Traditional Medicine. J. Pharmacopunct..

[116] Davidson P.M., Parish M.E. (1989). Methods for Testing the Efficacy of Food Antimicrobials. Food Technol..

[117] Odds F.C. (2003). Synergy, Antagonism, and What the Chequerboard Puts between Them. J. Antimicrob. Chemother.

[118] Mukherjee P.K., Banerjee S., Kar A. (2021). Molecular Combination Networks in Medicinal Plants: Understanding Synergy by Network Pharmacology in Indian Traditional Medicine. Phytochem. Rev..

[119] Mukherjee P.K., Harwansh R.K., Bahadur S., Biswas S., Kuchibhatla L.N., Tetali S.D., Raghavendra A.S. (2016). Metabolomics of Medicinal Plants—A Versatile Tool for Standardization of Herbal Products and Quality Evaluation of Ayurvedic Formulations. Curr. Sci..

[120] Villas-Boas S.G., Nielsen J., Smedsgaard J., Hansen M.A.E., Roessner-Tunali U. (2007). Metabolome Analysis: An Introduction.

[121] Wolfender J.-L., Marti G., Thomas A., Bertrand S. (2015). Current Approaches and Challenges for the Metabolite Profiling of Complex Natural Extracts. J. Chromatogr. A.

[122] Tafesh A., Najami N., Jadoun J., Halahlih F., Riepl H., Azaizeh H. (2011). Synergistic Antibacterial Effects of Polyphenolic Compounds from Olive Mill Wastewater. Evid.-Based Complement. Altern. Med..

[123] Kellogg J.J., Todd D.A., Egan J.M., Raja H.A., Oberlies N.H., Kvalheim O.M., Cech N.B. (2016). Biochemometrics for Natural Products Research: Comparison of Data Analysis Approaches and Application to Identification of Bioactive Compounds. J. Nat. Prod..

[124] Inui T., Wang Y., Pro S.M., Franzblau S.G., Pauli G.F. (2012). Unbiased Evaluation of Bioactive Secondary Metabolites in Complex Matrices. Fitoterapia.

[125] Gallo V., Matrorill P., Cafagna I., Nitti G.I., Latronico M., Longobardi F., Minoja A.P., Napoli C., Romito V.A., Schafer H. (2014). Effects of agronomical practices on chemical composition of table grapes evaluated by NMR spectroscopy. J. Food Compos. Anal..

[126] Hoerr V., Duggan G.E., Zbytnuik L., Poon K.K., Große C., Neugebauer U., Methling K., Löffler B., Vogel H.J. (2016). Characterization and prediction of the mechanism of action of antibiotics through NMR metabolomics. BMC Microbiol..

[127] Zhao X., Chen L., Wu J., He Y., Yang H. (2020). Elucidating antimicrobial mechanism of nisin and grape seed extract against *Listeria monocytogenes* in broth and on shrimp through NMR-based metabolomics approach. Int. J. Food Microbiol..

[128] More G.K., Vervoort J., Steenkamp P.A., Prinsloo G. (2022). Metabolomic profile of medicinal plants with anti-RVFV activity. Heliyon.

[129] Betancur L.A., Forero A.M., Vinchira-Villarraga D.M., Cárdenas J.D., Romero-Otero A., Chagas F.O., Pupo M.T., Castellanos L., Ramos F.A. (2020). NMR-based metabolic profiling to follow the production of anti-phytopathogenic compounds in the culture of the marine strain *Streptomyces* sp. PNM-9. Microbiol. Res..

[130] Guo C., He Y., Wang Y., Yung H. (2022). NMR-based metabolomic investigation on antimicrobial mechanism of *Salmonella* on cucumber slices treated with organic acids. Food Control.

[131] Caesar L.K., Kellogg J.J., Kvalheim O.M., Cech N.B. (2019). Opportunities and Limitations for Untargeted Mass Spectrometry Metabolomics to Identify Biologically Active Constituents in Complex Natural Product Mixtures. J. Nat. Prod..

[132] Martens H., Bruun S.W., Adt I., Sockalingum G.D., Kohler A. (2006). Pre-Processing in Biochemometrics: Correction for Path-Length and Temperature Effects of Water in FTIR Bio-Spectroscopy by EMSC. J. Chemom..

[133] Li P., AnandhiSenthilkumar H., Wu S., Liu B., Guo Z., Fata J.E., Kennelly E.J., Long C. (2016). Comparative UPLC-QTOF-MS-Based Metabolomics and Bioactivities Analyses of Garcinia Oblongifolia. J. Chromatogr. B.

[134] Caesar L.K., Kellogg J.J., Kvalheim O.M., Cech R.A., Cech N.B. (2018). Integration of Biochemometrics and Molecular Networking to Identify Antimicrobials in Angelica Keiskei. Planta Med..

[135] Nothias L.-F., Nothias-Esposito M., da Silva R., Wang M., Protsyuk I., Zhang Z., Sarvepalli A., Leyssen P., Touboul D., Costa J. (2018). Bioactivity-Based Molecular Networking for the Discovery of Drug Leads in Natural Product Bioassay-Guided Fractionation. J. Nat. Prod..

[136] Kohler I., Verhoeven A., Derks R.J., Giera M. (2016). Analytical pitfalls and challenges in clinical metabolomics. Bioanalysis.

[137] Han M.L., Liu X., Velkov T., Lin Y.W., Zhu Y., Creek D.J., Barlow C.K., Yu H.H., Zhou Z., Zhang J. (2019). Comparative Metabolomics Reveals Key Pathways Associated with the Synergistic Killing of Colistin and Sulbactam Combination against Multidrug-Resistant Acinetobacter baumannii. Front. Pharmacol..

[138] Cascante M., Marin S. (2008). Metabolomics and fluxomics approaches. Essays Biochem..

[139] Liu S.R., Peng X.X., Li H. (2019). Metabolic mechanism of ceftazidime resistance in *Vibrio alginolyticus*. Infect. Drug Resist..

[140] Choudhury A. (2022). Potential Role of Bioactive Phytochemicals in Combination Therapies against Antimicrobial Activity. J. Pharmacopunct..

[141] Evans S. (2008). Changing the Knowledge Base in Western Herbal Medicine. Soc. Sci. Med..

[142] Greco W.R., Bravo G., Parsons J.C. (1995). The Search for Synergy: A Critical Review from a Response Surface Perspective. Pharmacol. Rev..

[143] Cokol M., Chua H.N., Tasan M., Mutlu B., Weinstein Z.B., Suzuki Y., Nergiz M.E., Costanzo M., Baryshnikova A., Giaever G. (2011). Systematic Exploration of Synergistic Drug Pairs. Mol. Syst. Biol..

[144] Borisy A.A., Elliott P.J., Hurst N.W., Lee M.S., Lehar J., Price E.R., Serbedzija G., Zimmermann G.R., Foley M.A., Stockwell B.R. (2003). Systematic Discovery of Multicomponent Therapeutics. Proc. Natl. Acad. Sci. USA.

[145] Gertsch J. (2011). Botanical Drugs, Synergy, and Network Pharmacology: Forth and Back to Intelligent Mixtures. Planta Med..

[146] Bisson J., McAlpine J.B., Friesen J.B., Chen S.-N., Graham J., Pauli G.F. (2016). Can Invalid Bioactives Undermine Natural Product-Based Drug Discovery?. J. Med. Chem..

[147] Kellogg J.J., Paine M.F., McCune J.S., Oberlies N.H., Cech N.B. (2019). Selection and Characterization of Botanical Natural Products for Research Studies: A NaPDI Center Recommended Approach. Nat. Prod. Rep..

[148] Hyldgaard M., Mygind T., Meyer R.L. (2012). Essential Oils in Food Preservation: Mode of Action, Synergies, and Interactions with Food Matrix Components. Front. Microbiol..

[149] Schmidt B.M., Ribnicky D.M., Lipsky P.E., Raskin I. (2007). Revisiting the Ancient Concept of Botanical Therapeutics. Nat. Chem. Biol..

[150] Bucar F., Wube A., Schmid M. (2013). Natural Product Isolation—How to Get from Biological Material to Pure Compounds. Nat. Prod. Rep..

[151] Garvey M.I., Rahman M.M., Gibbons S., Piddock L.J.V. (2011). Medicinal Plant Extracts with Efflux Inhibitory Activity against Gram-Negative Bacteria. Int. J. Antimicrob. Agents.

[152] Roessner U., Bowne J. (2009). What Is Metabolomics All about?. Biotechniques.

[153] Liu M., Feng M., Yang K., Cao Y., Zhang J., Xu J., Hernández S.H., Wei X., Fan M. (2020). Transcriptomic and Metabolomic Analyses Reveal Antibacterial Mechanism of Astringent Persimmon Tannin against Methicillin-Resistant Staphylococcus Aureus Isolated from Pork. Food Chem..

[154] Ory L., Nazih E.-H., Daoud S., Mocquard J., Bourjot M., Margueritte L., Delsuc M.-A., Bard J.-M., Pouchus Y.F., Bertrand S. (2019). Targeting Bioactive Compounds in Natural Extracts—Development of a Comprehensive Workflow Combining Chemical and Biological Data. Anal. Chim. Acta.

[155] Allard P.-M., Péresse T., Bisson J., Gindro K., Marcourt L., Pham V.C., Roussi F., Litaudon M., Wolfender J.-L. (2016). Integration of Molecular Networking and In-Silico MS/MS Fragmentation for Natural Products Dereplication. Anal. Chem..

[156] Shaaban H.A. (2020). Essential Oil as Antimicrobial Agents: Efficacy, Stability, and Safety Issues for Food Application. Essential Oils-Bioactive Compounds, New Perspectives and Applications.

[157] Li J.W.-H., Vederas J.C. (2009). Drug Discovery and Natural Products: End of an Era or an Endless Frontier?. Science.

[158] Possemiers S., Bolca S., Verstraete W., Heyerick A. (2011). The Intestinal Microbiome: A Separate Organ inside the Body with the Metabolic Potential to Influence the Bioactivity of Botanicals. Fitoterapia.

[159] Stavropoulou E., Bezirtzoglou E. (2019). Predictive Modeling of Microbial Behavior in Food. Foods.

[160] Chabán M.F., Karagianni C., Joray M.B., Toumpa D., Sola C., Crespo M.I., Palacios S.M., Athanassopoulos C.M., Carpinella M.C. (2019). Antibacterial Effects of Extracts Obtained from Plants of Argentina: Bioguided Isolation of Compounds from the Anti-Infectious Medicinal Plant *Lepechinia Meyenii*. J. Ethnopharmacol..

[161] Candelaria-Dueñas S., Serrano-Parrales R., Ávila-Romero M., Meraz-Martínez S., Orozco-Martínez J., Ávila-Acevedo J.G., García-Bores A.M., Cespedes-Acuña C.L., Peñalosa-Castro I., Hernandez-Delgado T. (2021). Evaluation of the Antimicrobial Activity of Some Components of the Essential Oils of Plants Used in the Traditional Medicine of the Tehuacán-Cuicatlán Valley, Puebla, México. Antibiotics.

[162] Joray M.B., González M.L., Palacios S.M., Carpinella M.C. (2011). Antibacterial Activity of the Plant-Derived Compounds 23-Methyl-6-O-Desmethylauricepyrone and (*Z*,*Z*)-5-(Trideca-4,7-Dienyl)Resorcinol and Their Synergy with Antibiotics against Methicillin-Susceptible and -Resistant Staphylococcus Aureus. J. Agric. Food Chem..

[163] Joray M.B., Palacios S.M., Carpinella M.C. (2013). Understanding the Interactions between Metabolites Isolated from Achyrocline Satureioides in Relation to Its Antibacterial Activity. Phytomedicine.

[164] Wu P., Tang X., Jian R., Li J., Lin M., Dai H., Wang K., Sheng Z., Chen B., Xu X. (2021). Chemical Composition, Antimicrobial and Insecticidal Activities of Essential Oils of Discarded Perfume Lemon and Leaves (*Citrus Limon* (L.) Burm. F.) as Possible Sources of Functional Botanical Agents. Front. Chem..

[165] Stavropoulou E., Voidarou C.C., Rozos G., Vaou N., Bardanis M., Konstantinidis T., Vrioni G., Tsakris A. (2022). Antimicrobial Evaluation of Various Honey Types against Carbapenemase-Producing Gram-Negative Clinical Isolates. Antibiotics.

[166] Pincus D., Metten A. (2010). Nonlinear Dynamics in Biopsychosocial Resilience. Nonlinear Dyn. Psychol. Life Sci..

